# Exploratory toxicology studies of 2,3-substituted imidazo[1,2-*a*]pyridines with antiparasitic and anti-inflammatory properties

**DOI:** 10.1093/toxres/tfac046

**Published:** 2022-08-09

**Authors:** José Iván Serrano-Contreras, María Estela Meléndez-Camargo, Yazmín Karina Márquez-Flores, Martha Patricia Soria-Serrano, María Elena Campos-Aldrete

**Affiliations:** Departamento de Química Orgánica, Escuela Nacional de Ciencias Biológicas, Instituto Politécnico Nacional, Prolongación de Carpio y Plan de Ayala s/n, Col. Santo Tomas, C.P. 11340, Delegación Miguel Hidalgo, Ciudad de México, México; Departamento de Farmacia, Escuela Nacional de Ciencias Biológicas, Instituto Politécnico Nacional, Av. Wilfrido Massieu 399, Unidad Profesional Adolfo López Mateos, Col. Nueva Industrial Vallejo, C.P. 07738, Delegación Gustavo A. Madero, Ciudad de México, México; Departamento de Farmacia, Escuela Nacional de Ciencias Biológicas, Instituto Politécnico Nacional, Av. Wilfrido Massieu 399, Unidad Profesional Adolfo López Mateos, Col. Nueva Industrial Vallejo, C.P. 07738, Delegación Gustavo A. Madero, Ciudad de México, México; Departamento de Farmacia, Escuela Nacional de Ciencias Biológicas, Instituto Politécnico Nacional, Av. Wilfrido Massieu 399, Unidad Profesional Adolfo López Mateos, Col. Nueva Industrial Vallejo, C.P. 07738, Delegación Gustavo A. Madero, Ciudad de México, México; Departamento de Farmacia, Escuela Nacional de Ciencias Biológicas, Instituto Politécnico Nacional, Av. Wilfrido Massieu 399, Unidad Profesional Adolfo López Mateos, Col. Nueva Industrial Vallejo, C.P. 07738, Delegación Gustavo A. Madero, Ciudad de México, México; Departamento de Química Orgánica, Escuela Nacional de Ciencias Biológicas, Instituto Politécnico Nacional, Prolongación de Carpio y Plan de Ayala s/n, Col. Santo Tomas, C.P. 11340, Delegación Miguel Hidalgo, Ciudad de México, México

**Keywords:** imidazo[1,2-*a*]pyridines, multivariate analysis, partial least squares, off-target effect, vehicle effect

## Abstract

**Background:**

Trichomoniasis and amoebiasis are neglected diseases and still remain as a global health burden not only for developing countries, from where are endemic, but also for the developed world. Previously, we tested the antiparasitic activity of a number of imidazo[1,2-*a*]pyridine derivatives (IMPYs) on metronidazole-resistant strains of *Entamoeba Hystolitica* (HM1:IMSS), and *Trichomonas Vaginalis* (GT3). Their anti-inflammatory activity was also evaluated.

**Objective:**

The present work is a part of a project whose aim is to find new alternatives to standard treatments for these maladies, and to address the current concern of emerging resistant parasite strains. Here we report a non-clinical study focused on exploratory toxicology assays of seven IMPYs that showed the best antiparasitic and/or anti-inflammatory properties.

**Methods:**

Acute, and subacute toxicity tests were carried out. After 14-day oral treatment, liver and kidney functionality assays in combination with chemometric methods were implemented to detect hepatic and/or kidney damage.

**Results:**

Some compounds produced off-target effects. Vehicle effects were also detected. However, no signs of hepatic or renal toxicity were observed for any IMPY.

**Conclusion:**

These compounds can continue non-clinical evaluations, and if possible, clinical trials as new candidates to treat trichomoniasis and amoebiasis, and inflammatory diseases. Further studies are also needed to fully elucidate a proposed dual effect that may exert these molecules against trichomoniasis and amoebiasis, which may also signify a novel mechanism of action to treat these infections.

## Graphical Abstract

**Graphical Abstract:**
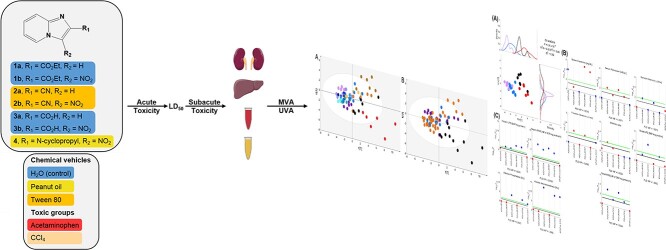

## Introduction

*Trichomonas vaginalis* is a flagellated protozoan parasite that causes the most prevalent nonviral sexually transmitted infection worldwide, which can cause serious consequences to both the individual and community health, where the majority of infections occur in developing countries.^1–4^ The 2016 global prevalence estimates of this infection in women and men were 5.3% (95% uncertainty interval [UI]: 4.0–7.2) and 0.6% (95% UI: 0.4–0.9), respectively; which correspond to the total of 110.4 million cases. The global incidence rate estimated was 156.0 million (95% UI: 103.4–231.2 million) cases in women and men aged 15–49 years, where low-income countries, territories, and areas had the highest prevalence. This estimate corresponds to an average of >420,000 new infections per day.^5^^,^^6^

About 50% of women with trichomoniasis will be asymptomatic, but symptomatic cases generally present urogenital conditions ranging from pruritis, dyspareunia, and vaginal discharge causing severe vaginal, ectocervical, prostatic, and urethral inflammations, and this infection is also linked with sterility, pelvic inflammatory disease, and cervical cancers.^1^^,^^2^ Some of the most serious complications occur when an infection is present during pregnancy, which can lead to premature rupture of the placental membranes, resulting in premature labor and low birth-weight babies.^1^^,^^7^ In the case of men, *T. vaginalis* secretes a protein homologous to human macrophage migration inhibitory factor (MIF) which has proinflammatory properties, and with combination of chronic inflammation, trichomoniasis can have an impact on the host immune system regulation and can play a role in the increased risk of prostate cancer.^3^^,^^8^ The *T. vaginalis* infection is also implicated in increasing the risk of acquisition and transmission of HIV.^1^^,^^5^^,^^6^

Amoebiasis is a parasitic disease caused by *Entamoeba histolytica*, one of the most significant extracellular enteropathogens worldwide. Even 162 years after its first detection, amoebiasis still remains as a global health problem. According to the World Health Organization, amoebiasis is responsible for an estimated 35–50 million cases of symptomatic diseases and approximately 40,000–100,000 deaths annually.^2^^,^^9–13^ In the case of Mexico, >8.8 million cases of amoebiasis were reported in its National Epidemiological Surveillance System between 2000 and 2010.^13^ The majority of *E. histolytica* infections are asymptomatic; only about 10%–20% progress to develop symptomatic infection that could lead to a severe disease with amebic colitis (inflammatory diarrhea), amebic liver abscess, and/or metastatic invasion, where >50% of cases with severe colitis die.^2^^,^^8^^,^^12–14^ Inadequate sanitary conditions in endemic regions and the presence of highly pathogenic strains of *E. histolytica* may combine to sustain a high incidence of both intestinal amoebiasis and amoebic liver abscess.^15^ Thus, this parasitosis is a major health and social issue not only in the developing world but also among people from the developed world, such as immigrants and returning travelers from endemic areas, in particular those volunteering as missionary workers or doing other volunteering work affecting general as well as military populations.^2^^,^^8–13^^,^^15^

Amoebiasis could be easily avoided by adopting basic hygiene habits and by having access to a toilet and tap water. Rural populations are the most disadvantaged, where 8 out of 10 people are still lacking basic drinking water services, and 7 out of 10 people are still lacking basic sanitation live in rural areas. Preventing the spread of diseases is not as easy as it should be, as without safe sanitation, handwashing with soap and water, diseases spread rapidly.^11^^,^^16^^,^^17^ In addition, every day, >700 children die from diarrhea linked to unsafe water, sanitation, and hygiene. Diarrhea is a major contributor to childhood mortality and morbidity in the developing world, causing an estimated 2.5 million deaths each year and long-term effects on growth and cognitive function.^9^^,^^13^^,^^18^ Small children are capable of infecting entire families.^9^^,^^10^ It has been reported that 2 out of 7 sites studied across sub-Saharan Africa and South Asia, *E. histolytica* was 1 of the top 10 causative agents of moderate to severe diarrhea in children under the age of 5 years. The *E. histolytica* diarrhea was also associated with a relatively greater risk of death across all these sites and was the enteric pathogen with the highest hazard ratio for death in the second year of life.^12^

Metronidazole and tinidazole are the mainstay treatment for both trichomoniasis and amoebiasis. Metronidazole is relatively cheap and tinidazole is generally more expensive.^1^^,^^7^^,^^12^ Tinidazole has a longer half-life and is better tolerated, but metronidazole is as effective at clearing parasites.^12^ Although metronidazole is relatively effective and well tolerated, metronidazole resistance has been reported in 2.5–9.6% of trichomoniasis cases,^1^ which has been implicated in an increasing number of refractory cases.^4^ It has also been reported that resistance to metronidazole occurs in 4–10% of cases of vaginal trichomoniasis and that resistance to tinidazole in 1%.^7^ The *T. vaginalis* infection does not lead to long-term immunity and reinfection can readily occur. Additionally, trichomoniasis is often asymptomatic and as such goes untreated, creating reservoirs of *T. vaginalis* which allow the disease to spread within the community.^1^ In addition, these drugs produce several side effects. For instance, metronidazole may cause nausea, headache, anorexia, vomiting (due to its metallic taste), heartburn, constipation, diarrhea, peripheral neuropathy, cerebellar ataxia, and disulfiram-like reaction with alcohol. Both drugs have been related to central and peripheral nervous system adverse effects. Due to these issues, the treatment could be abandoned and the infection cannot be properly eradicated which in turn may accelerate antimicrobial resistance.^11–13^ Despite this, a number of new drug candidates have been investigated, and there are still few alternatives to standard therapy, making emerging resistant trichomoniasis and amoebiasis a global concern.^1^^,^^4^^,^^7^^,^^9^^,^^12–15^^,^^17^

It is noteworthy to mention that we also explored their anti-inflammatory activity, as inflammatory responses by the host is a part of the pathogenesis of both *Entamoeba hystolitica* and *T. vaginalis*. Recent studies have helped to deepen the understanding about the pathogenesis of amoebiasis, which appears to derive from parasite cytotoxic activity, damaging inflammatory response, and tissue invasion. Many pathogenic protozoa, including *E. hystolitica* and *T. vaginalis*, secrete a protein homolog of the human proinflammatory cytokine macrophage MIF.^3^^,^^8^^,^^14^ Thus, the IMPYs reported here may have both properties, however, further studies are needed to confirm this dual action in vivo and to continue the process of research and development for these molecules.

Regarding only the treatment of inflammation, inflammation diseases currently represent a major global cause of morbidity related to the modern lifestyle. Inflammation is a defensive response developed by the host against the invasion by foreign bodies, including not only parasites but also bacteria and viruses. However, when uncontrolled, it leads to a wide range of acute and chronic debilitating diseases, including psoriasis, immune-inflammatory ailments, neoplastic transformations, gout, rheumatoid arthritis, multiple sclerosis, diabetes, inflammatory bowel disease, cancer, Alzheimer’s disease, and atherosclerosis, along with pulmonary, autoimmune, and cardiovascular diseases. The mainstay treatment for inflammatory diseases is the steroidal and nonsteroidal anti-inflammatory drugs (NSAIDs). However, the use of corticosteroids leads to hypertension, hyperglycemia, osteoporosis, and growth arrest; and the chronic use of NSAIDs is reported to cause severe adverse effects like gastrointestinal, cardiovascular, and renal toxicities. Furthermore, many chronic diseases manifest due to presence of low-grade sustained inflammation; and the toxicity and recurrence of symptoms on discontinuation is a major problem related to current treatments.^19^

Taking into account all the aforementioned makes a high priority the development of new effective and safer drugs to treat these maladies. Accordingly, to make a contribution on this regard, our group has been involved in the research of new candidates, where initially a number of imidazo[1,2-*a*]pyridine (IMPY) derivatives were synthesized and tested *in vitro* against resistant strains of *E. hystolitica* (HM1:IMSS) and *T. vaginalis* (GT3), also a highly pathogenic strain), with the exemption of the cyclopropyl derivative.^20–22^ In an additional study, we also evaluated the *in vivo* and *in vitro* anti-inflammatory activity of these compounds, including the cyclopropyl derivative.^23^^,^^24^ Here, we report a preclinical follow-up study focused on toxicity tests to detect hepatic and/or kidney damage of 7 IMPYs that showed to be the best candidates with antiparasitic and/or anti-inflammatory properties, and in the case of the latter, with no damage to the gastroduodenal tract. These compounds proved to be essentially nontoxic, and the changes that they produced in the liver and kidney were most related to off-target and vehicle effects.

## Materials and methods

### Chemistry

The chemical compounds ethyl imidazo[1,2-*a*]pyridine-2-carboxylate (1a), ethyl 3-nitroimidazo[1,2-*a*]pyridine-2-carboxylate (1b), imidazo[1,2-*a*]pyridine-2-carbonitrile (2a) and 3-nitroimidazo[1,2-*a*]pyridine-2-carbonitrile (2b) were prepared according to previously reported procedures.^20^^,^^21^^,^^25^^,^^26^

The quantities were scaled up in the range of grams to enable early evaluation in exploratory toxicology studies. The compounds were identified through their physical constants as well as spectroscopic data. IR spectra were obtained on a Perkin Elmer Spectrum 2000 FT-IR Spectrometer. Melting points were determined on an Electrothermal IA9000 melting point apparatus and were uncorrected. ^1^H and ^13^C NMR spectra were recorded in either deuterated chloroform (CDCl_3_) or deuterated dimethyl sulfoxide (DMSO-d_6_). Spectra were obtained on either a Varian Mercury NMR 300 MHz or a Varian NMR system 500 spectrometer.

The compounds imidazo[1,2-*a*]pyridine-2-carboxylic acid (3a), 3-nitroimidazo[1,2-*a*]pyridine-2-carboxylic acid (3b), and 2-(*N*-cyclopropyl)-3-nitroimidazo[1,2-*a*]pyridine (4) included in the present study were taken from the same batches reported previously during the investigation of anti-inflammatory activity.^23^^,^^24^

### Animal handling

Adult female Wistar rats (200 ± 20 g of body weight (b.w.) and adult female NIH mice (25 ± 3 g of b.w.) were kept in polycarbonate cages (Allentown Inc., Allentown, NJ, US) and were allowed to acclimatize themselves for 7 days under controlled environmental conditions (temperature, 22–24 °C; relative humidity, 50–55%; a 12/12 h light cycle, with access to a standard rodent diet (PMI Nutrition International, LLC. Rodent Laboratory Chow 5001, Brentwood, MO, United States) and water *ad libitum*. The drug vehicles used in the present investigation were as follows: water for 1a-b, 3a-b, and acetaminophen (APAP); Tween 80 for 2a-b; peanut oil (PO) for 4 and carbon tetrachloride (CCl_4_). All treatments were administrated orally (p.o.) according to the scheme mentioned below.

All experimental procedures were carried out in accordance with the guidelines stipulated by Mexican laws and regulations for the care and use of laboratory animals, in the Seventh Title of the Regulations of the General Law of Health in the Matter of Health Research and the Mexican Official Standard (NOM-062-ZOO-1999). The protocol was also approved by the Internal Bioethics Committee (CEI-ENCB-019-2016).

### Toxic models

#### Acute systemic repeated-dose toxicity

Acute toxicity tests were performed in order to measure the median lethal dose (LD_50_). After acclimatization, mice were randomly allocated to ten groups (*n* = 4), including the control group (e.g. water, Tween 80, or PO) and the experimental groups, each with a single dose of one of the IMPYs at 4 levels of dose (0.5, 1.0, 2.0, and 4.0 g/kg b.w.).

To assess the LD_50_, the animals were under observation for 3 h after dosing and then were monitored for 14 days to record the number of survivors every day. Animals were food deprived overnight prior to administration of the chemical compounds or vehicles, and food was returned 4 h after dosing. Animals had access to water *ad libitum* throughout the study.^27^^,^^28^

#### Subacute repeated-dose toxicity

For toxicity tests, acclimatized rats were randomly allocated to 12 groups (*n* = 12 for the control and *n* = 6 for the rest groups). A 14-day oral treatment, with 10% of the LD_50_ of 1 of the IMPYs, was applied to each of the 7 groups. Meanwhile, the aforementioned vehicles (dose volume 2 mL/kg b.w.) were administered to 3 groups. Additionally, 1 group was administered CCl_4_ (1:1, v/v in PO; 2.5 mL/kg b.w.) and another APAP (1 g/kg b.w.) was administered once per day (p.o.) during 3 days. Upon completion of the 14-day treatments and before sample collection, the bladders of animals were emptied by gentle pressure on the lower part of the abdomen to induce urination. The animals were then housed in individual metabolic cages to collect urine for a period of 6 h and then the volume was recorded. Afterward, blood samples were obtained and the serum was separated by centrifugation at 1,620 × *g* for 10 min at room temperature.

### Urine and serum analysis

#### Glucose and protein urinary excretion

Serum and urine glucose concentration were calculated using GOD-PAP kits (RANDOX, Laboratories Ltd, UK). Urinary protein was quantified using the Bradford method.^29^

#### Glomerular filtration rate

Serum and urinary creatinine were determined by the Jaffe method. With this value, evaluation was made of the fractional excretion of sodium (FES), fractional excretion of potassium (FEP), and fractional excretion of glucose.^30^^,^^31^ The GFR was calculated from clearance of endogenous creatinine using the conventional equation:

$$ \mathrm{GFR}={C}_{\mathrm{Cr}}=\left({U}_{\mathrm{Cr}}\times \varphi\ \right)/{S}_{\mathrm{Cr}}, $$

where GFR (*C*_Cr_) is the creatinine clearance rate (mL/min); *U*_Cr_ and *S*_Cr_ are the creatinine concentrations (mg/dL) in urine and serum, respectively; and φ is the urinary flow rate (UFR) (mL/min). To avoid the error from the tubular secretion of creatinine found in males, only female animals were used.

#### Water and electrolyte balance

Osmolality of serum and urine were assessed (in triplicate) by using a vapor pressure osmometer (Wescor 5500, Logan, United States). U/S ratio as well as osmolar (*C*_Osm_) and free water (${C}_{{\mathrm{H}}_2\mathrm{O}}$) clearances were calculated via conventional equations:

$$ \mathrm{U}/\mathrm{S}\ \mathrm{ratio}={U}_{\mathrm{Osm}}/{S}_{\mathrm{Osm}}, $$

$$ {C}_{\mathrm{Osm}}=\left({U}_{\mathrm{Osm}}\times \varphi\ \right)/{S}_{\mathrm{Osm}}, $$

$$ {C}_{{\mathrm{H}}_2\mathrm{O}}=\varphi -{C}_{\mathrm{Osm}}, $$

where *U*_Osm_ and *S*_Osm_ are the osmolality values (mOsm/Kg) in urine and serum, respectively, and φ is the UFR (mL/min).

The levels of sodium and potassium were measured in urine and serum using a flame photometer (Corning 400, Medfield, United States). Calculation was made of the clearance, fractional excretion, and filtered load of sodium (FLS), filtered load of potassium (FLP), and filtered load of glucose (FLG) using the conventional equations:

$$ {C}_X=\left({U}_X\times \varphi\ \right)/{S}_X, $$

$$ {\mathrm{FE}}_X=\left({C}_X/{C}_{\mathrm{Cr}}\right)\times 100, $$

$$ {\mathrm{FL}}_X={C}_{\mathrm{Cr}}\times{S}_X, $$

where *C_X_* is the clearance rate of X (mL/min), *C*_Cr_ is the creatinine clearance rate (mL/min), *U_X_* and *S_X_* are the serum and urinary concentrations of X, respectively, φ is the UFR (mL/min), FE_X_ is the fractional excretion (%) of X, and FL_X_ is the filtered load of X in μEq/min (Na^+^ or K^+^) or mg/min (glucose).

#### Alanine aminotransferase and alkaline phosphatase activity

The activities of serum alanine aminotransferase (ALT) and alkaline phosphatase (ALP) were measured with standard kits (RANDOX, Laboratories Ltd, UK).

### Tissue analysis

#### Active tubular secretion of organic acids

*p*-Aminohippuric acid (PAH) was used as an indicator of the renal secretory pathway of organic anions. Kidneys were extracted and immediately submerged in Ringer solution (pH = 7.4 and mOsm = 290 ± 10 mOsm/kg) under constant bubbling with O_2_/CO_2_ (95/5%). The kidneys were decapsulated and slices of the kidney cortex were obtained, which were then incubated in Ringer solution containing PAH (1 mM) for 1 h at 25 °C under constant bubbling with O_2_/CO_2_ (95/5%). The levels of PAH were quantified with the Bratton-Marshall method and results were expressed as the PAH tissue/medium ratio (PAH t/m ratio).^31^^,^^32^

#### Hepatic and renal lipid peroxidation

Lipid peroxidation (LPO) was evaluated by the formation of lipid-soluble fluorescence, as previously described. Briefly, kidney and liver tissues were homogenized in 3 and 5 mL of phosphate buffer (0.2 M, pH = 7.4), respectively. A volume of 1 mL (kidney) or 0.8 mL (liver) of the homogenates was added to 7 mL of chloroform-methanol (2:1, v/v), and after 15 s of vortex mixing, the resulting mixture was cooled using an ice bath for 30 min to allow for phase separation. The fluorescence of the organic phase was measured using a Shimadzu fluorometer (Japan; λ_ex_ = 370 nm; λ_em_ = 430 nm), whose sensitivity was adjusted to 140 fluorescence units by using 1 μg/mL of quinine sulfate in 50 mM H_2_SO_4_. The results were expressed as relative fluorescence units per milligram of protein.^33^

#### Quantification of hepatic and renal reactive oxygen species

Reactive oxygen species (ROS) were measured by formation of 2′,7′-dichlorofluorescein (DCF). An aliquot of 10 μL (kidney) or 5 μL (liver) of the homogenates was added to 1,940 or 1,945 μL of TRIS:HEPES (18:1) and was incubated in the presence of 50 μL of 2′,7′-dichlorofluorescein diacetate for 1 h at 37 °C. In order to quench the reaction, the mixture was chilled using an ice bath. The fluorescence was measured using a Shimadzu fluorometer (Japan; λ_ex_ = 488 nm; λ_em_ = 525 nm). The results were expressed as nM DCF formed per mg protein/h.^33^

#### Quantification of protein

The protein concentration in homogenates was measured by the Bradford method.^29^

### Statistical analysis

Multivariate data analysis (MVA) was performed using SIMCA software (v. 13.0; Umetrics, Sweden). Principal component analysis (PCA) was carried out in order to observe trends among all the groups included in the study. PCA is an unsupervised approach which is able to reduce the dimension of a multivariate data set into fewer variables, namely principal components (PC) or latent variables. PCs are linear combinations of the original variables, but orthogonal to each other, whereby the first PC projects as much variation in the data as possible, and the second PC shows the second largest variation and so forth.

Partial least squares (PLS) models were built according to the vehicle used for a given compound or set of compounds. The control group was included in all of the models. PLS is a supervised approach that also reduces a multidimensional space into fewer variables but relates a data matrix (*X*) to a response matrix (*Y*) to each other via a linear multivariate model. The PLS models were constructed to investigate the statistical relationship between the hepatic and renal functionality parameters (*X*) and the treatments (*Y* = 0, 1, . . ., *n* + 1). In order to give the same prior importance in the analysis, *X* variables were mean centered and scaled to unit variance before the PLS modeling, and to avoid overfitting, each model was built with 2 components. The models were validated by 7-fold crossvalidation (CV), CV-analysis of variance (ANOVA), and permutation test with 500 permutations. CV is an approach for internal predictive validation which is used to determine the predictable Y-variation, denoted by Q^2^Y. CV-ANOVA provides a significance metric for single-Y by using the residuals of CV, considering a significant model with a *P*-value ≤ 0.05.^34–37^

#### Construction of models

Five models were built. Model 1, the “toxic model,” included only the data set from toxic agents and their respective vehicles (*Y* = 0, 1, 10, 11). Model 2 analyzed the data set from the control, 1a-b, and 3a-b (*Y* = 0, 3, 4, 7, 8). Model 3 was built with the data set from the control, tween 80, and 2a-b (*Y* = 0, 2, 5, 6). For model 4, the control, peanut oil, and 4 were included (*Y* = 0, 1, 9). Model 5, the “vehicle model,” included only the data from the control and the 2 vehicles (*Y* = 0, 1, 2).

#### Variable selection

The variable influence on projection (VIP) values from the PLS models were used to select important variables. VIP summarizes the importance of an *X*-variable in the model with respect to *Y* and *X*. VIP values > 1 are the most relevant for explaining *Y*. Therefore, a cutoff threshold value of VIP ≥ 1 was used to select the most relevant variables (MRVs) from the PLS models.^34^^,^^35^^,^^38^

#### Univariate data analysis

Univariate analysis (UVA) was performed for each MRV by comparing the classes in all the PLS models. The corresponding data set was imported into MatLab (R2015b, The MathWorks, Natick, MA) to perform one-way ANOVA, followed by the post hoc Bonferroni adjustment to compensate for multiple comparisons. The variables with VIP ≥ 1 and statistical significance via UVA were considered for biological interpretation.

## Results

### Synthesis

**Fig. 1:**
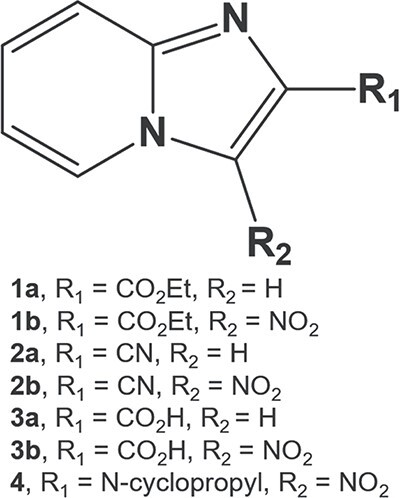
Imidazo[1,2-*a*]pyridines evaluated in in vivo preclinical toxicology studies.

The synthesis of compounds 1a-b and 2a-b was scaled-up according to previous methods,^19^^,^^20^^,^^24^^,^^25^ where good yields were achieved. The corresponding characterization data of each compound is reported in Supplementary Table S1. The compounds of the preclinical trial portfolio used in the present study are illustrated on Fig. 1.

### Acute systemic repeated-dose toxicity

According to the mice LD_50_ values (g/kg b.w.), compounds 2a (0.794) and 2b (1.606) with a carbonitrile moiety at the C-2 position of the imidazole ring were more toxic than 1a (3.175) and 1b (>4.000), which are functionalized at the same position with an ethoxycarbonyl group. In both cases, compounds 1b and 2b, with a nitro group at the C-3 position, were less toxic than their corresponding 1a and 2a derivatives. Regarding the LD_50_ values observed for 3a (>2.000 g/kg b.w.), 3b (>2.000 g/kg b.w.), and 4 (1 g/kg b.w.), these molecules were approximately at the midpoint of toxicity compared to the rest of the tested IMPYs. Nevertheless, the 7 compounds were allocated to hazard categories 4 and 5 based on oral acute toxicity expressed as (approximate) LD_50_ values, as stated in the Globally Harmonized System of Classification and Labelling of Chemicals (UN, 2019).^39^ Therefore, they were classified as having relatively low toxicity (Supplementary Table S2).

### Subacute repeated-dose toxicity

The PCA applied to the entire data set (*X*) gave a two-component model explaining 79% (*R*^2^) and predicting 68% (*Q*^2^) of the data variation. The score plot reveals a main cluster at the bottom-right of the score plot, which corresponds to the model toxicants. For peanut oil and 2b, group clustering trends were also observed. However, for the rest of groups, the separation was less evident in comparison with the control group (Supplementary Fig. S1). In general, all the PLS models were significant according to CV, CV-ANOVA, and permutation tests. The summary of the data set is reported in Supplementary Table S3.

From the “toxic model” (model 1) and its corresponding one-way ANOVA analyses on MRVs indicated important changes derived from the inherent toxicity of APAP and CCl_4_. APAP decreased serum potassium, freewater clearance, and the PAH t/m ratio and increased osmolar clearance, renal and hepatic ROS and LPO compared to the control group. CCl_4_ increased renal and hepatic ROS and LPO and serum ALP and ALT in comparison to the control and PO. In this model, it was also observed that PO increased serum creatinine compared to the control group (Supplementary Fig. S2, Supplementary Table S4).

**Fig. 2:**
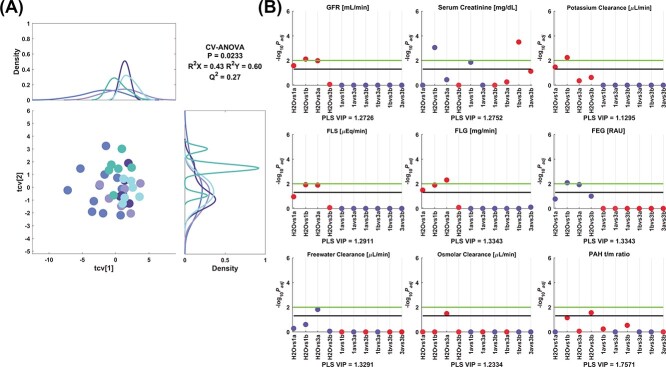
Model 2 corresponding to the comparison between control and 1a-b/3a-b. A) PLS crossvalidated scores plot along with the corresponding density plots for both components derived from kernel density estimates and scaled to a maximum estimated value of 1. Color code: (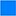) control, (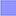) 1a, (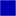) 1b, (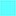) 3a, and (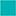) 3b. B) Manhattan plots of the significant variables corresponding to the renal functionality parameters. The color code is according to the difference between the estimated group means, more than (blue) or less than (red) the control, 1a, 1b, or 3a. Cutoffs: black (−log_10_(.05) = 1.3), green (−log_10_(.01) = 2.0). Key: PLS VIP, VIP from the PLS model; FEG, fractional excretion of glucose; RAU, rationalized arcsine transform. Adjusted *P*-values (*P*_adj_) are reported in Supplementary Table S5.

The model 2 corresponding to 1a-b and 3a-b showed different changes, where only renal effects were observed, as depicted on Fig. 2. GFR was lower for 1a, 1b, and 3a and serum creatinine level was greater only for 1b in comparison to the control group. Potassium clearance was decreased by 1a-b compared to the control. FLS was decreased for the 1b and 3a treatments in comparison with the control group. FLG was reduced by 1a, 1b and 3a, and FEG increased with 1b and 3a compared to control values. Freewater clearance and osmolar clearance were greater and lower for 3a than the control, respectively. The 3b decreased the PAH t/m ratio versus control values. Moreover, serum creatinine was higher for 1b than for 1a and 3b.

**Fig. 3:**
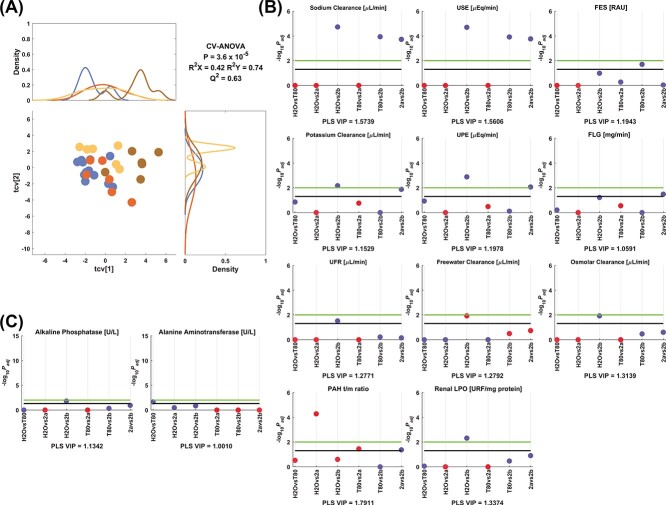
Model 3 corresponding to the comparison between control, tween 80, and 2a-b. A) PLS crossvalidated scores plot along with the corresponding density plots for both components derived from kernel density estimates and scaled to a maximum estimated value of 1. Color code: (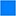) control, (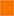) tween 80, (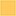) 2a, and (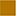) 2b. Manhattan plots of the significant variables corresponding to the B) renal and C) hepatic functionality parameters. The color code is according to the difference between the estimated group means, more than (blue) or less than (red) the control, tween 80, or 2a. Cutoffs: black (−log_10_(0.05) = 1.3), green (−log_10_(0.01) = 2.0). Adjusted *P*-values (*P*_adj_) are reported in Supplementary Table S6.

The clustering trend observed in the crossvalidated scores plot and the direction of its corresponding MRVs from the model 3 can be observed in Fig. 3. Sodium clearance and urinary sodium excretion (USE) was higher for 2b than control, tween 80, and 2a. FES was also increased by 2b in comparison with tween 80. Potassium clearance and urinary potassium excretion (UPE) were increased by 2b compared to the control and 2a, and FLG was only increased by 2b versus 2a. UFR was increased by 2b in comparison with the control group. Freewater clearance and osmolar clearance were lower and greater for 2b than the control, respectively. Renal LPO and ALP were greater for 2b than the control, and tween 80 increased ALT in comparison to control values. The treatment with 2a also decreased the PAH t/m ratio versus control, Tween 80, and 2b.

**Fig. 4:**
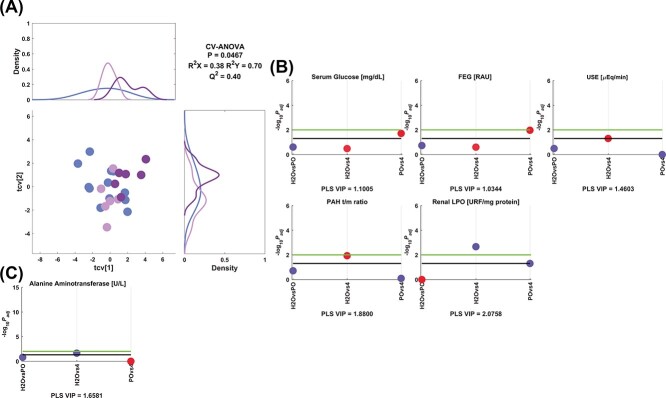
Model 4 corresponding to the comparison between control, peanut oil and 4. A) PLS crossvalidated scores plot along with the corresponding density plots for both components derived from kernel density estimates and scaled to a maximum estimated value of 1. Color code: (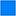) control, (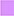) peanut oil, and (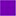) 4. Manhattan plots of the significant variables corresponding to the B) renal and C) hepatic functionality parameters. The color code is according to the difference between the estimated group means, more than (blue) or less than (red) the control or peanut oil. Cutoffs: black (−log_10_(.05) = 1.3), green (−log_10_(.01) = 2.0). Adjusted *P*-values (*P*_adj_) are reported in Supplementary Table S7**.**

In Fig. 4, the output of the model 4 is depicted, where it is observed that serum glucose and FEG were diminished with compound 4 only when compared to PO and that USE was reduced in comparison to control values. Renal LPO and ALT were greater with 4 than the control. The treatment with 4 also reduced the PAH t/m ratio compared to the control group.

**Fig. 5:**
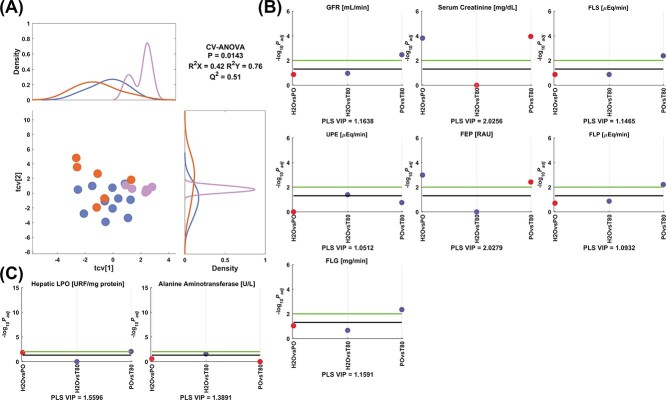
Model 5 corresponding to the comparison between control, peanut oil, and tween 80. A) PLS crossvalidated scores plot along with the corresponding density plots for both components derived from kernel density estimates and scaled to a maximum estimated value of 1. Color code: (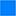) control, (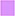) peanut oil, and (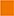) tween 80. Manhattan plots of the significant variables corresponding to the B) renal and C) hepatic functionality parameters. The color code is according to the difference between the estimated group means, more than (blue) or less than (red) the control or peanut oil. Cutoffs: black (−log_10_(.05) = 1.3), green (−log_10_(.01) = 2.0). Adjusted *P*-values (*P*_adj_) are reported in Supplementary Table S8.

For the “vehicle model” (Fig. 5), it was observed that concomitant to the increment of serum creatinine and FEP, PO reduced hepatic LPO in comparison to the control and tween 80. Moreover, ALT and UPE were increased by tween 80 compared to the control values. GFR, FLS, FLP, and FLG were increased by tween 80 versus PO.

## Discussion

Pharmaceutical research and development is a long, complex, and expensive process with a low success rate of new drugs outputs. One of the obvious targets for improving the efficiency of this process is the first stage of discovery consisting of *in vivo* exploratory toxicology studies carried out with rodent models. These preliminary studies are comprised of acute and subacute toxicity tests aimed at evaluating possible development-limiting drawbacks, including toxicity and possible off-target effects, the latter of which could be related to side effects or pharmacological activity. In order to detect organ-specific toxicity, the inclusion of at least one dosage group is recommended, as well as weeks of repeated dosing when using only traditional end points (e.g. serum chemistry).^19^^,^^40^^,^^41^

Models of hepatotoxicity are based on certain chemicals (e.g. CCl_4_ and _D_-galactosamine) and drugs (e.g. APAP, thioacetamide, azathioprine, and doxorubicin). The main hepatotoxic mechanisms are related to any bioactivation that stimulates an excessive production of ROS and thus the depletion of antioxidant enzymes (e.g. glutathione [GSH]) and/or the perturbation of bile acid transport. This causes oxidative stress and LPO which in turn leads to mitochondrial injury and depletion of ATP and finally to cell death by necrosis. As several enzymes are produced in the liver and are normally distributed within the cells of this organ, high levels of serum ALT and ALP have been taken as sensitive biomarkers of hepatotoxicity. Furthermore, hepatic damage is also suspected with the advent of numerous biochemical changes related to oxidative stress, such as free radical formation and LPO.^40^^,^^42^

Nephrotoxicity, on the other hand, is induced by some NSAIDs, angiotensin-converting enzyme inhibitors, angiotensin receptor blockers, antibiotics, and antifungal agents as well as antiretroviral and anticancer drugs. The major mechanisms for drug-induced nephrotoxicity include changes in glomerular hemodynamic, tubular cell alterations (i.e. proximal tubule damage), inflammation, crystal nephropathy, rhabdomyolysis, and thrombotic microangiopathy. Nephrotoxicity can be diagnosed by measuring, in blood and urine tests, the level of serum creatinine, the glomerular filtration rate (GFR), and the proteinuria and electrolyte-water balance.^43^

In order to facilitate this first stage of discovery in drug design and development, one possibility is a more efficient and effective method for the analysis of data collected. In this sense, MVA approach reduces dimensionality, handles many variables and few observations, many observations and few variables, and extracts information from all data simultaneously. Two common MVA techniques are the projection methods PCA and PLS, which are tools for extracting and visualizing trends, groupings, and unique objects in data sets of varying sizes. PLS has proved to be a powerful tool for finding relationships between descriptor matrices and biological responses in order to predict passive intestinal absorption of some drugs *in vivo* in humans, or to generate QSAR models as a tool to optimize potency and ADME properties.^35–37^ In this way, discovery of patterns related with toxicological or vehicle effects is possible through PLS models built from a set of hepatic and renal functionality parameters.

The present study was based on a targeted approach on renal and hepatic effects because the liver and kidney are the primary routes of biotransformation and excretion of xenobiotics. With the presence of foreign substances, these 2 organs represent the major control systems that maintain homeostasis. In addition, the liver is a key part of the first pass effect. Therefore, these 2 organs can be regarded as the major target organs for xenobiotic-induced toxicity.^42^^,^^43^ Our research was also based on the core strategy in any preclinical evaluation, where the detection of any drug-induced liver injury (DILI) manifestations is key. In our particular case, in addition to detect *in vivo* flags of hepatotoxicity, we also covered nephrotoxicity assessment as some molecules under study have affinity to cyclooxygenase (COX) isoenzymes, COX-1, and COX-2, which are involved in kidney physiology.

In this context, we first validated the applicability of the renal and hepatic functionality parameters measured to detect flags of hepatotoxicity and/or nephrotoxicity by using known chemical toxicity models, namely CCl_4_ and APAP, which exert different mechanisms of toxicity. Their corresponding data give a PLS model that denotes a toxicological pattern of both toxicants, hence referred as “toxic model,” which was used as a template for biological interpretation in relation to the effects produced by the 7 IMPYs and their vehicles. It is well known that acute ingestion of CCl_4_ causes kidney damage in association with liver failure. This toxicant is biotransformed into trichloromethyl and trichloromethyl peroxyl radicals that are able to bind to proteins and lipids of the cell membrane, which leads to oxidative stress and LPO.^42^ Accordingly, CCl_4_ was indeed found to provoke renal and hepatic oxidative stress and LPO as well as necrosis and cholestasis in the liver. Furthermore, as ALP is also expressed on the brush border membranes of proximal tubular cells, high serum level of this enzyme has been related to tubular damage.^44^ APAP, an analgesic and antipyretic drug, induces hepatotoxicity through a different bioactivation pathway than that of CCl_4_. Following biotransformation, APAP yields an electrophilic metabolite, *N*-acetyl-*p*-benzoquinoneimine (NAPQI) that undergoes GSH conjugation. GSH levels can be depleted with toxic doses of APAP, leaving free NAPQI to covalently bind to proteins. The resulting oxidative damage can lead to hepatocyte and renal injury and necrosis, as illustrated in the “toxic model” regarding APAP.^41^^,^^42^

Based on the acute oral treatment with each of the 7 tested IMPYs, it was then possible to adjust the doses in order to explore the effects under a subacute oral treatment (i.e. 10% of the LD_50_). The results were consistent, as they showed that these compounds are essentially nontoxic. In the subacute tests, some changes in renal and hepatic physiology were mostly related to vehicle effects or to inherent pharmacological properties (as described below). These changes were detected in the “problem models” that were built to evaluate only the effects produced by the vehicles and IMPYs. Concomitantly, the “vehicle model” was built to define only the variables most closely related to the vehicle effects (not necessarily related to toxicity). Hence, the presence or absence of toxic and/or vehicle effects could be observed by classifying the IMPYs with their respective PLS models.

Numerous xenobiotics and xenobiotic-induced metabolites possess the carboxylic acid moiety, which may be metabolized *in vitro* and *in vivo* to oxidative metabolites, acyl glucuronides, and/or S-acyl-CoA thioesters (CoA conjugates). Therefore, these molecules have the potential of forming reactive metabolites that mediate adverse drug reactions in humans. Since acyl glucuronides and their isomers are unstable in physiological conditions, they perhaps mediate liver and kidney injury by means of covalent adducts with GSH, proteins and DNA via transacylation or rapid acyl migration. This group of metabolites represents the most important route of metabolism for a number of carboxylic acid-containing drugs (including NSAIDs) and metabolites, whose corresponding glucuronides can be excreted into the bile, where they may also disrupt bile acid homeostasis and cause cholestasis. Additionally, the CoA conjugates are about 40- to 70-fold more reactive toward GSH and proteins than the corresponding acyl glucuronide. It is possible that these conjugates also interfere with β-oxidation, disrupt mitochondrial function, or deplete the CoA, carnitine, and GSH reserves of cells. CoA conjugates can thus contribute to the hepatotoxicity and nephrotoxicity of xenobiotics in humans.

Accordingly, several NSAIDs with a carboxylic acid moiety have been terminated in clinical trials, withdrawn from the market after being launched, or restricted in their use through black box warnings due to toxicity (cholestatic or hepatocellular DILI or renal toxicity). Such NSAIDs include ibuprofen, ibufenac, diclofenac, fenclozic acid, zomepirac, bromfenac, benoxaprofen, and tolmetin.^45–48^ In this context, ibuprofen and tolmetin are still in the market as oral treatments (the intended route of administration for our candidates), where the former is a common over-the-counter product and the latter a prescribed drug. Aspirin is another instance of a safe drug that can produce acyl glucuronides, as known elsewhere. In our case, and despite the concern on the IMPYs with a carboxylic acid moiety (3a-b) or with a group that can produce it (1a-b), these molecules herein caused no organ-specific toxicity in liver or kidney. However, they gave rise to off-target effects, mainly in renal physiology.

Prostaglandins are important in maintaining a normal GFR through vasodilatation of afferent vessels. Since COX is responsible for maintaining adequate levels of prostaglandins, the selective and nonselective inhibition of this enzyme by NSAIDs reduces the GFR. COX-1 and COX-2 are involved not only in regulating the GFR but also the equilibrium of sodium, potassium, and water. Hence, NSAIDs can lead to nephrotoxicity.^43^^,^^49^ Given that glomerular hemodynamic were disrupted by 1a, 1b, and 3a, they possibly act as nonselective anti-inflammatory drugs.^23^^,^^24^ Accordingly, the changes in renal processing of glucose, potassium, and sodium caused by 1a, 1b, and 3a may also be related to the inhibition of COXs expressed in collecting ducts, arteriolar endothelial cells, mesangial cells, epithelial cells of the Bowman capsule, macula densa, epithelial cells lining the ascending loop of Henle, and the medullary interstitial cells of the renal papillae.^49^^,^^50^

The effects produced by the IMPYs and their vehicles were observed. For instance, Tween 80 is a hydrophilic nonionic surfactant used in oral and other pharmaceutical formulations. This compound is a polyethylene sorbitol monoester with various fatty acids, the main one being oleic acid. Others include palmitoleic and palmitic acids. After oral administration in rats, the ester bond of Tween 80 is hydrolyzed by pancreatic lipase, at a rate of 100%, within the digestive tract, thereby releasing fatty acid and polyoxyethylene sorbitan. The free fatty acids (FFAs) are then absorbed from the digestive tract, metabolized, and excreted. Since Tween 80 is a source of FFAs and at the same time increases the intestinal absorption of fatty acids, oral administration of this compound is likely to give rise to an accumulation of lipids in the liver. *In vitro* assays showed that Tween 80 modified mitochondrial respiration to a degree proportional to its concentration. In a subchronic toxicity study in mice fed with this compound, cytoplasmic accumulation of lipids was observed in hepatocytes. Therefore, the high levels of ALT brought about by Tween 80 are perhaps related to these mechanisms as well as to the surfactant properties of this compound and the physicochemical properties of cell membranes.^51–54^ For instance, Tween 80 can modify the intestinal absorption of electrolytes or the permeability of the erythrocyte membrane. Consequently, these mechanisms could possibly be related to the tween 80-induced changes in the renal processing of potassium.^52–54^ This vehicle can then exert effects additional to those produced by 2a and 2b in the physiology of the liver and kidney.

The nitrile group is not particularly electrophilic toward free nucleophiles (not even to GSH). In fact, in most cases, it is not readily metabolized and is likely excreted unchanged. In cases of drug metabolism, sites remote from the nitrile group can undergo phase I and/or II biotransformation, while the nitrile group remains unmodified and no release of cyanide from aromatic or fully substituted carbons is found.^55^ Hence, 2a and 2b may be less likely to give rise to toxic effects.

Although 2b showed diverse effects on water and electrolyte balance and high serum ALP, these changes were probably related to the effect of the vehicle. After oral administration, Tween 80 is able to promote the absorption of these substances and likely that of their major metabolites by inhibition of the permeability glycoprotein (P-gp) efflux pump in the intestinal epithelial cells. This effect is similar to that of di-(2-ethylhexyl) phthalate. Thus, Tween 80 perhaps enhances absorption and increases the risk of toxicity of 2b. Besides the surfactant properties of Tween 80, the rise in the level of ALP is possibly caused by the disruption of lipid metabolism in the liver which this compound can provoke.^51^^,^^52^^,^^56^

PO has also been used as a pharmaceutical excipient. Its major fats take the form of triacylglycerol, with c.a. 87% unsaturated and 13% saturated fat.^57^^,^^58^ The elevated levels of serum creatinine by PO might be related to tubular damage, which would also affect the renal processing of potassium. Accordingly, the repeated administration of PO probably increases serum levels of triglycerides or FFAs and this in turn should impair the function of glomerular capillary membranes and thus reduce the surface area for filtration. However, these effects were not associated with proteinuria or renal oxidative stress, indicating nonnephrotoxic activityper se.

Another moiety to consider is *N*-cyclopropylamine, which is found in numerous drugs and drug candidates. Many of these undergo cytochrome P450-catalyzed *N*-dealkylation to yield reactive intermediates that can form protein adducts.^59^ The renal LPO as well as the elevation of serum ALT caused by 4 may be attributed to this effect.

On the other hand, organic anion transporters (OATs) expressed at the basolateral membrane of proximal tubules are related to the renal elimination of endobiotics as well as xenobiotics. PAH is a well-known substrate for rat OATs and has been used as a marker for renal OATs.^60^ Hence, 2a, 3b, and 4 may compete for PAH secretion, and consequently, these could have more affinity for rat OATs than PAH. Moreover, PAH uptake is augmented by prostaglandin E_2_, whose reduction by NSAIDs downregulates OATs in renal proximal tubules. Since COX-1 and COX-2 are involved in OAT function and 3b and 4 have affinity for these enzymes,^23^^,^^24^ the decrease in PAH secretion is possibly related to this mechanism as well as to the competition for OATs transport,^61^ which can be proved by the reduction of PAH t/m ratio produced by APAP.

Subacute toxicity test was done using 10% of the LD_50_, considering that the main objective was to use a dose that does not lead to death but could modify the renal and hepatic parameters evaluated as observed for some compounds tested. However, additional tests are required to fully determine the subacute toxicity of multiple doses for them.

## Conclusion

The discovery and development of new drugs is a difficult, expensive, and long process where success is not granted. It becomes more challenging where the new treatments are aimed at neglected diseases such as amoebiasis and trichomoniasis. In the case that research groups from developing countries are interested on developing new drugs to treat their endemic maladies, this challenge is exacerbated due to limited resources and the low-level or no interaction between academia and industry. This makes them unable to improve the tests and conclude this process to know whether one of their candidates reach the market or not, and in a success case, whether it is affordable or not for the underprivileged who are the most affected by these parasitoses. In this context, our group, as many others, regards these infections not as neglected diseases but as a major health problem that still remains affecting not only the developing countries but also the developed world, hence our contribution to find new treatments. Accordingly, the 7 best IMPYs with antiparasitic and anti-inflammatory properties were essentially not hepato-nephrotoxic. Off-target effects produced by some of them were also observed, where the vehicle may be involved. PO and Tween 80 were not inert to hepatic and renal physiology but instead exerted either unique or synergistic effects with the coadministered compounds. Potential toxicities should be considered for the compounds 2b and 4. Since this is a first-time study to report toxicity tests of the 7 IMPYs, the present research allows to propose a possible range of doses for further nonclinical and/or clinical studies as new candidates to treat amoebiasis and trichomoniasis and inflammatory diseases.

Some of our candidates may have the proposed dual action exerted by the same compound (i.e. antiparasitic and anti-inflammatory) which in turn may have a better therapeutic outcome than the current treatments for both trichomoniasis and amoebiasis which only exert antiparasitic activity. However, as part of preclinical strategy, further studies are needed to fully elucidate this mechanism of action.

## Authors’ contributions

Conceptualization was by J.I.S.-C., M.E.M.-C., and M.E.C.-A. Investigation was by J.I.S.-C., Y.K.M.-F., M.P.S.-S., and M.E.C.-A. Writing—review and editing was done by J.I.S.-C., M.E.M.-C., Y.K.M.-F., and M.E.C.-A. J.I.S.-C. took care of the formal analysis, software, validation, writing—original draft preparation, and visualization. M.E.M.-C. was responsible for methodology, supervision, and funding acquisition. M.E.C.-A. was in charge of resources.

## Supplementary Material

Supplementary_Material_tfac046
