# Supplementary material for: Exploratory toxicology studies of 2,3-substituted imidazo[1,2-a]pyridines with antiparasitic and anti-inflammatory properties
Source: Toxicol Res (Camb). 2022 Aug 9;11(5):730–42. doi: 10.1093/toxres/tfac046 (PMC9618103; doi:10.1093/toxres/tfac046)
Supplement: Supplementary_Material_tfac046 [file supplementary_material_tfac046.docx]

# Supporting Information

**Title: Exploratory toxicology studies of 2,3-substituted imidazo[1,2-*a*]pyridines with antiparasitic and anti-inflammatory properties.**

José Iván Serrano-Contreras^a,b,c^*, María Estela Meléndez-Camargo^b^, Yazmín Karina Márquez-Flores^b^_,_ Martha Patricia Soria-Serrano^b^, María Elena Campos-Aldrete^a^

^a^Departamento de Química Orgánica, Escuela Nacional de Ciencias Biológicas, Instituto Politécnico Nacional, Prolongación de Carpio y Plan de Ayala s/n, Col. Santo Tomas C.P. 11340, Delegación Miguel Hidalgo, Ciudad de México, México

^b^Departamento de Farmacia, Escuela Nacional de Ciencias Biológicas, Instituto Politécnico Nacional, Av. Wilfrido Massieu s/n, Esq. Manuel Stampa, Unidad Profesional Adolfo López Mateos, C.P. 07738, Delegación Gustavo A. Madero, Ciudad de México, México

^c^Department of Metabolism, Digestion and Reproduction, Division of Systems Medicine, Imperial College London, South Kensington, London, SW7 2AZ, UK.

*- corresponding author:

Email: [jserranoc@yahoo.com](mailto:jserranoc@yahoo.com), j.serrano-contreras@imperial.ac.uk

^a,b^Sponsor affiliation

^c^Present affiliation


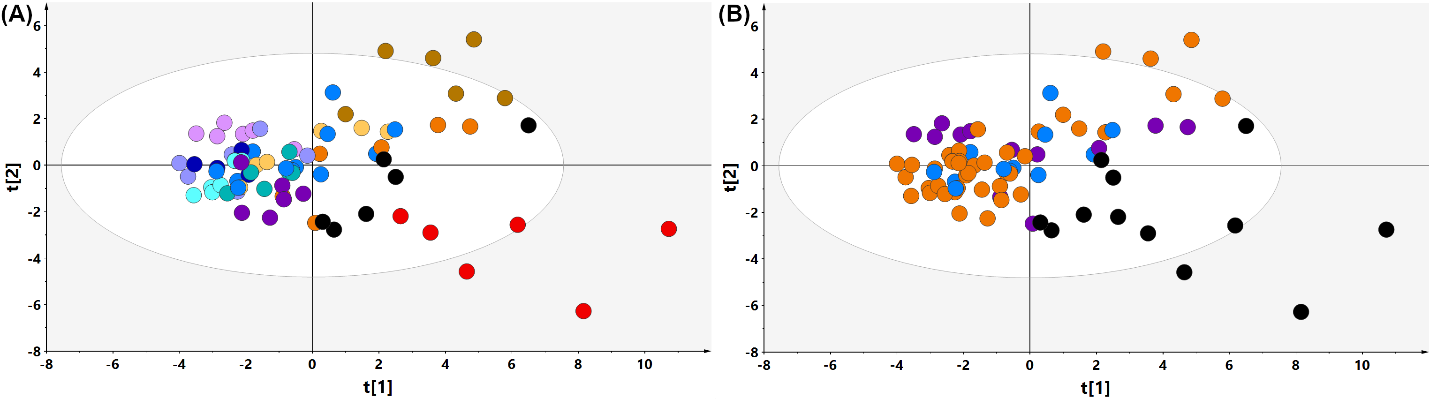


**Figure S1.** Same PCA scores plot depicting all the groups included in the present study. A) Color code: () control, () tween 80, () peanut oil, () 1a, () 1b, () 2a, () 2b, () 3a, () 3b, () 4, () CCl_4_, () APAP. B) Color code: () control, () imidazo[1,2-*a*]pyridines, () drug vehicles, () toxicants. R^2^X = 0.79, Q^2^ = 0.68.


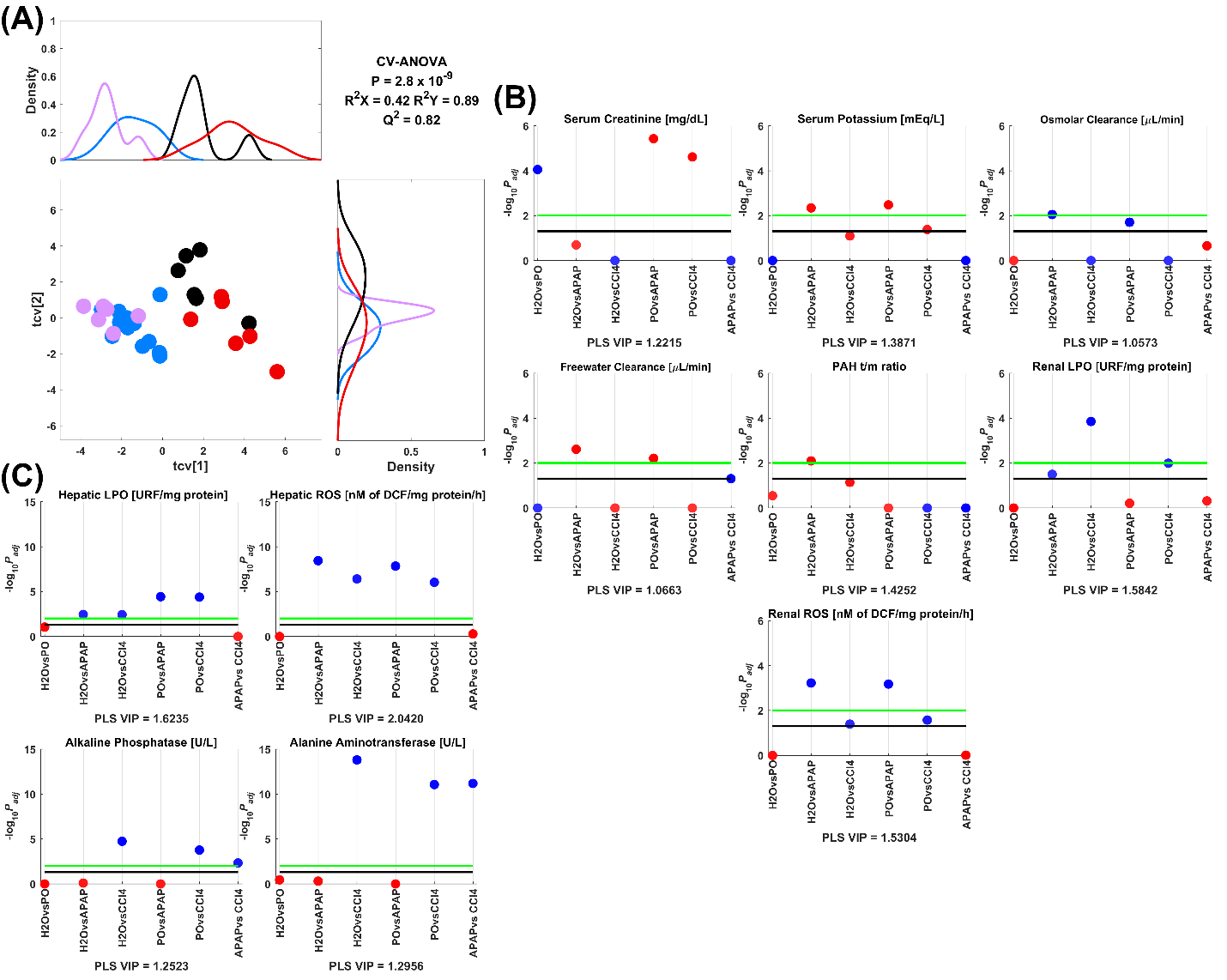


**Figure S2.** Proof-of-the-concept model with toxicants. A) PLS cross-validated scores plot along with the corresponding density plots for both components derived from kernel density estimates and scaled to a maximum estimated value of 1. Color code: () control, () peanut oil, () CCl_4_, () APAP. Manhattan plots of the significant variables corresponding to the renal (B) and hepatic (C) functionality parameters. The color code is according to the difference between the estimated group means, more than (blue) or less than (red) the control, PO or APAP. Cut-offs: black (-log_10_(0.05) = 1.3), green (-log_10_(0.01) = 2.0). Key: PLS VIP, variable influence on projection from the PLS model; PAH t/m ratio, PAH tissue to medium ratio. Adjusted *P*-values (*P*_adj_) are reported in **Supplementary Table S4**.

**Table S1.** Summary of physical and chemical data, and IR and NMR peak assignments for **1a-b** and **2a-b**

| **Molecule** | **Physical constants and spectroscopic data^a^** |
| --- | --- |
| **1a** | 65.57 g of white needle crystals, 77.9%, mp: 177 ºC. IR (KBr cm^-1^): υ 1725 (C=O), 1269 (C−O), 3087 (C−H_ar_), 2981 (C−H_al_). ^1^H NMR (DMSO-d_6_, 300 MHz): δ 1.39 (t, 3H), 4.46 (q, 2H), 7.58 (t, 1H), 7.93 (d, 1H), 8.04 (t, 1H), 9.01 (d, 1H), 9.103 (s, 1H); ^13^C{^1^H} NMR (DMSO-d_6_, 75 MHz): δ 14.08, 62.28, 113.22, 117.52, 119.21, 126.77, 129.63, 134.84, 141, 158.68. |
| **1b** | 27.48 g of yellow needle crystals, 46.0% (two steps), mp: 106 ºC. IR (KBr cm^-1^): υ 1736 (C=O), 1241 (C−O), 3074 (C−H_ar_), 2987 (C−H_al_), υ_s_ 1376 (NO_2_), υ_as_ 1536 (NO_2_). ^1^H NMR (DMSO-d_6_, 300 MHz): δ 1.36 (t, 3H), 4.44 (q, 2H), 7.54 (t, 1H), 7.86 (t, 1H), 7.98 (d, 1H), 9.27 (d, 1H); ^13^C{^1^H} NMR (DMSO-d_6_, 75 MHz): δ 13.82, 62.38, 118.26, 118.42, 128.09, 132.34, 139.94, 144.60, 162.19. |
| **2a** | 3.28 g of brown crystals, 46.2% (three steps), mp: 176 ºC. IR (KBr cm^-1^): υ 2234 (C≡N), 3105 (C−H_ar_). ^1^H NMR (CDCl_3_, 300 MHz): δ 6.98 (t, 1H), 7.35 (t, 1H), 7.64 (d, 1H), 8.1 (s, 1H), 8.18 (d, 1H); ^13^C{^1^H} NMR (CDCl_3_, 75 MHz): δ 114.58, 114.68, 118.41, 119.25, 125.98, 127.23, 145.34. |
| **2b** | 11.52 g of yellow crystals, 36.4% (four steps), mp: 163 ºC. IR (KBr cm^-1^): υ_s_ 1358 (NO_2_), υ_as_ 1539 (NO_2_), υ 2249 (C≡N), 3140 (C−H_ar_). ^1^H NMR (CDCl_3_, 500 MHz): δ 7.47 (t, 1H), 7.79 (t, 1H), 7.94 (d, 1H), 9.41 (d, 1H); ^13^C{^1^H} NMR (CDCl_3_, 125 MHz): δ 11.65, 118.84, 119.55, 120.77, 127.21, 131.84, 145.31. |
| ^a^As reported in the literature (López-Martínez et al., 2012; Lombardino, 1965; Arias et al., 2006). | |

**Table S2.** Summary of the acute toxicological profile of evaluated compounds.

| Compound | R_1_ | R_2_ | Oral LD_50_ | Category^a^ | Hazard statement^a^ |
| --- | --- | --- | --- | --- | --- |
|  |  |  | [g/kg b.w.] |  |  |
| **1a** | CO_2_Et | H | 3.175 | 5 | May be harmful if swallowed |
| **1b** | CO_2_Et | NO_2_ | >4.000 | 5 | May be harmful if swallowed |
| **2a** | CN | H | 0.794 | 4 | Harmful if swallowed |
| **2b** | CN | NO_2_ | 1.606 | 4 | Harmful if swallowed |
| **3a** | CO_2_H | H | > 2.000 | 5 | May be harmful if swallowed |
| **3b** | CO_2_H | NO_2_ | > 2.000 | 5 | May be harmful if swallowed |
| **4** | *N*-cyclopropyl | NO_2_ | 1.000 | 4 | Harmful if swallowed |

Water was used as vehicle for **1a-b** and **3a-b**, tween 80 for **2a-b** and peanut oil for **4**. ^a^According to the literature [39].

**Table S3.** Summary of variables considered in the present study.

| **Name** | **Groups** | | | | | | | | | | | |
| --- | --- | --- | --- | --- | --- | --- | --- | --- | --- | --- | --- | --- |
|  | **Control** | **Tween 80** | **PO** | **CCl_4_** | **APAP** | **1a** | **1b** | **2a** | **2b** | **3a** | **3b** | **4** |
|  | **(n = 12)** | **(n = 6)** | **(n = 6)** | **(n = 6)** | **(n = 6)** | **(n = 6)** | **(n = 6)** | **(n = 6)** | **(n = 6)** | **(n = 6)** | **(n = 6)** | **(n = 6)** |
| **Serum Glucose [mg/dL]** | 119.61± 5.21 | 124.51 ± 17.45 | 135.72 ± 9.15 | 103.17 ± 4.35 | 128.13 ± 2.96 | 112.39 ± 4.92 | 121.27 ± 7.88 | 103.46 ± 3.16 | 126.47 ± 5.03 | 80.56 ± 2.31 | 121.37 ± 8.03 | 104.90 ± 1.91 |
| **Urinary Glucose Excretion [ng/min]** | 163.69± 42.74 | 257.75 ± 39.58 | 125.38 ± 36.08 | 265.11 ± 89.54 | 87.10 ± 19.92 | 96.54 ± 29.03 | 141.87 ± 18.46 | 136.89 ± 32.82 | 109.06 ± 24.61 | 92.91 ± 12.35 | 262.45 ± 19.41 | 37.92 ± 13.79 |
| **Glucose Clearance [μL/min]** | 0.14 ± 0.04 | 0.23 ± 0.05 | 0.10 ± 0.04 | 0.28 ± 0.11 | 0.07 ± 0.2 | 0.08 ± 0.02 | 0.12 ± 0.02 | 0.13 ± 0.03 | 0.09 ± 0.02 | 0.12 ± 0.01 | 0.22 ± 0.02 | 0.04 ± 0.01 |
| **Filtered Load of Glucose [mg/min]** | 1.19± 0.21 | 1.86 ± 0.35 | 0.37 ± 0.07 | 4.64 ± 1.71 | 1.35 ± 0.27 | 0.39 ± 0.17 | 0.31 ± 0.04 | 0.90 ± 0.29 | 2.29 ± 0.33 | 0.22 ± 0.03 | 0.74 ± 0.09 | 0.81 ± 0.20 |
| **Fractional Excretion of Glucose [%]** | 0.02 ± 0.01 | 0.01 ± 6.9E-4 | 0.03 ± 0.01 | 0.02 ± 0.01 | 0.01 ± 1.9E-3 | 0.04 ± 0.01 | 0.05 ± 3.9 E-3 | 0.02 ± 3.9E-3 | 0.01 ± 1.8E-3 | 0.05 ± 0.01 | 0.04 ± 3.7E-3 | 0.01 ± 1.7E-3 |
| **Urinary Flow Rate [μL/min]** | 4.49 ± 0.76 | 6.30 ± 1.79 | 4.40 ± 0.57 | 6.59 ± 1.39 | 6.19 ± 1.15 | 4.17 ± 1.25 | 4.54 ± 0.76 | 6.44 ± 1.39 | 8.29 ± 1.67 | 2.20 ± 0.50 | 3.75 ± 0.75 | 3.43 ± 0.59 |
| **Urinary Protein Excretion [μg/min]** | 1.93 ± 0.49 | 1.16 ± 0.21 | 1.19 ± 0.25 | 12.15 ± 4.49 | 0.94 ± 0.24 | 0.56 ± 0.10 | 0.57 ± 0.07 | 0.65 ± 0.13 | 0.86 ± 0.20 | 0.69 ± 0.14 | 1.18 ± 0.23 | 1.64 ± 0.29 |
| **Glomerular Filtration Rate [mL/min]** | 0.96 ± 0.16 | 1.66 ± 0.39 | 0.29 ± 0.07 | 4.30 ± 1.47 | 1.05 ± 0.21 | 0.34 ± 0.14 | 0.25 ± 0.03 | 0.86 ± 0.28 | 1.81 ± 0.26 | 0.28 ± 0.04 | 0.63 ± 0.08 | 0.77 ± 0.19 |
| **Serum Creatinine [mg/dL]** | 0.73 ± 0.09 | 0.53 ± 0.06 | 1.81 ± 0.28 | 0.45 ± 0.13 | 0.28 ± 0.03 | 0.80 ± 0.14 | 1.38 ± 0.07 | 0.66 ± 0.13 | 0.56 ± 0.08 | 1.05 ± 0.05 | 0.57 ± 0.12 | 0.87 ± 0.20 |
| **Serum Potassium [mEq/L]** | 5.40 ± 0.32 | 5.29 ± 0.21 | 5.71 ± 0.14 | 4.27 ± 0.25 | 3.78 ± 0.22 | 6.57 ± 0.32 | 6.74 ± 0.39 | 6.03 ± 0.31 | 5.79 ± 0.31 | 6.83 ± 0.54 | 6.69 ± 0.40 | 5.50 ± 0.34 |
| **Potassium Clearance [μL/min]** | 118.34 ± 16.23 | 206.04 ± 38.13 | 119.72 ± 18.80 | 174.79 ± 23.80 | 128.42 ± 41.18 | 51.13 ± 8.86 | 37.00 ± 6.18 | 108.98 ± 29.92 | 241.82 ± 39.60 | 73.73 ± 16.94 | 67.82 ± 13.97 | 69.96 ± 21.14 |
| **Fractional Excretion of Potassium [%]** | 15.61 ± 2.98 | 15.18 ± 4.52 | 52.48 ± 11.98 | 6.86 ± 2.06 | 12.50 ± 3.12 | 22.26 ± 6.39 | 15.58 ± 3.10 | 13.55 ± 1.23 | 13.47 ± 1.95 | 27.89 ± 6.73 | 11.63 ± 2.44 | 17.06 ± 10.83 |
| **Urinary Potassium Excretion [μEq/min]** | 0.61 ± 0.08 | 1.09 ± 0.21 | 0.68 ± 0.11 | 0.73 ± 0.10 | 0.47 ± 0.14 | 0.34 ± 0.06 | 0.24 ± 0.03 | 0.64 ± 0.16 | 1.38 ± 0.21 | 0.50 ± 0.12 | 0.43 ± 0.07 | 0.35 ± 0.08 |
| **Filtered Load of Potassium [μEq/min]** | 5.10 ± 0.92 | 8.80 ± 2.07 | 1.70 ± 0.43 | 17.22 ± 5.29 | 3.99 ± 0.80 | 2.69 ± 1.19 | 1.70 ± 0.21 | 5.07 ± 1.44 | 10.21 ± 1.12 | 1.98 ± 0.44 | 4.15 ± 0.53 | 4.34 ± 1.12 |
| **Serum Sodium [mEq/L]** | 137.94 ± 12.10 | 144.63 ± 4.03 | 139.78 ± 2.50 | 139.08 ± 1.63 | 138.58 ± 2.37 | 162.39 ± 5.03 | 155.42 ± 3.63 | 142.94 ± 3.88 | 141.65 ± 15.47 | 148.12 ± 3.02 | 144.61 ± 3.41 | 137.20 ± 2.08 |
| **Sodium Clearance [μL/min]** | 3.41 ± 0.76 | 3.21 ± 0.61 | 1.77 ± 0.69 | 5.21 ± 1.00 | 3.23 ± 1.43 | 1.82 ± 0.53 | 2.19 ± 0.37 | 3.61 ± 0.67 | 13.93 ± 3.09 | 1.14 ± 0.32 | 2.36 ± 0.42 | 0.74 ± 0.19 |
| **Fractional Excretion of Sodium [%]** | 0.43 ± 0.11 | 0.22 ± 0.04 | 0.44 ± 0.17 | 0.21 ± 0.06 | 0.27 ± 0.09 | 0.74 ± 0.22 | 0.85 ± 0.09 | 0.55 ± 0.13 | 0.88 ± 0.29 | 0.53 ± 0.16 | 0.38 ± 0.05 | 0.18 ± 0.10 |
| **Urinary Sodium Excretion [μEq/min]** | 0.50 ± 0.11 | 0.47 ± 0.10 | 0.24 ± 0.09 | 0.72 ± 0.13 | 0.46 ± 0.21 | 0.29 ± 0.08 | 0.34 ± 0.05 | 0.51 ± 0.09 | 1.87 ± 0.39 | 0.17 ± 0.05 | 0.34 ± 0.06 | 0.10 ± 0.03 |
| **Filtered Load of Sodium [μEq/min]** | 141.61 ± 22.73 | 242.92 ± 61.59 | 40.27 ± 9.01 | 592.11 ± 199.71 | 147.38 ± 31.27 | 64.29 ± 26.43 | 39.31 ± 4.28 | 124.49 ± 40.90 | 267.24 ± 51.35 | 40.07 ± 7.62 | 90.75 ± 12.03 | 103.86 ± 25.22 |
| **Serum Osmolality [mOsm/kg]** | 294.72 ± 1.70 | 315.09 ± 11.86 | 291.20 ± 1.49 | 305.77 ± 3.41 | 295.68 ± 1.57 | 293.75 ± 1.88 | 294.04 ± 0.88 | 291.74 ± 1.34 | 288.93 ± 1.51 | 304.73 ± 1.82 | 293.74 ± 2.06 | 295.85 ± 2.12 |
| **Osmolar Clearance [μL/min]** | 15.70 ± 2.24 | 21.00 ± 4.90 | 14.75 ± 1.69 | 20.84 ± 3.89 | 33.72 ± 6.87 | 11.15 ± 2.57 | 10.82 ± 1.29 | 20.21 ± 3.83 | 27.75 ± 5.17 | 6.13 ± 1.22 | 11.27 ± 1.83 | 11.30 ± 1.58 |
| **Freewater Clearance [μL/min]** | -11.20 ± 1.68 | -14.70 ± 3.19 | -10.36 ± 1.13 | -14.25 ± 2.65 | -27.53 ± 5.86 | -6.99 ± 1.38 | -6.28 ± 0.69 | -13.77 ± 2.46 | -19.46 ± 3.50 | -3.93 ± 0.82 | -7.52 ± 1.21 | -7.87 ± 1.02 |
| **U/S Osm ratio** | 4.00 ± 0.69 | 3.81 ± 0.61 | 3.41 ± 0.09 | 3.27 ± 0.37 | 5.44 ± 0.60 | 3.02 ± 0.25 | 2.54 ± 0.20 | 3.30 ± 0.22 | 3.44 ± 0.10 | 3.18 ± 0.61 | 3.21 ± 0.39 | 3.34 ± 0.17 |
| **PAH t/m ratio** | 2.64 ± 0.15 | 2.05 ± 0.38 | 2.21 ± 0.15 | 2.08 ± 0.11 | 1.89 ± 0.10 | 2.51 ± 0.13 | 2.00 ± 0.13 | 1.07 ± 0.11 | 2.11 ± 0.20 | 2.25 ± 0.10 | 1.92 ± 0.23 | 1.92 ± 0.10 |
| **Renal Lipoperoxidation [URF/mg protein]** | 0.25 ± 0.02 | 0.32 ± 0.05 | 0.32 ± 0.07 | 0.61 ± 0.07 | 0.46 ± 0.06 | 0.28 ± 0.03 | 0.38 ± 0.07 | 0.30 ± 0.02 | 0.47 ± 0.03 | 0.29 ± 0.03 | 0.44 ± 0.09 | 0.57 ± 0.10 |
| **Renal ROS [nM de DCF/mg protein/h]** | 175.75 ± 12.54 | 201.45 ± 24.91 | 140.40 ± 22.27 | 333.27 ± 74.29 | 421.62 ± 56.57 | 161.18 ± 27.84 | 227.17 ± 21.01 | 171.33 ± 22.04 | 225.78 ± 56.22 | 159.65 ± 14.96 | 184.33 ± 42.31 | 169.56 ± 16.66 |
| **Alkaline Phosphatase [U/L]** | 90.81 ± 13.62 | 127.72 ± 18.86 | 94.14 ± 28.53 | 328.19 ± 61.36 | 151.97 ± 11.19 | 93.14 ± 11.91 | 101.41 ± 16.30 | 104.44 ± 19.76 | 192.21 ± 38.07 | 93.76 ± 13.08 | 56.63 ± 6.67 | 81.42 ± 17.37 |
| **Alanine Aminotransferase [U/L]** | 30.93 ± 3.84 | 49.70 ± 6.43 | 42.52 ± 3.02 | 127.80 ± 4.08 | 41.47 ± 4.80 | 22.22 ± 1.69 | 30.10 ± 2.99 | 42.68 ± 2.04 | 44.90 ± 2.46 | 25.62 ± 1.27 | 32.76 ± 5.37 | 47.54 ± 3.10 |
| **Hepatic Lipoperoxidation [URF/mg protein]** | 0.69 ± 0.04 | 0.75 ± 0.02 | 0.44 ± 0.09 | 1.06 ± 0.11 | 1.06 ± 0.06 | 0.75 ± 0.04 | 0.74 ± 0.07 | 0.71 ± 0.04 | 0.64 ± 0.10 | 0.79 ± 0.03 | 0.80 ± 0.03 | 0.81 ± 0.08 |
| **Hepatic ROS [nM de DCF/mg protein/h]** | 322.51 ± 39.45 | 267.75 ± 37.11 | 212.43 ± 37.83 | 1434.82 ± 121.35 | 1740.28 ± 231.55 | 317.94 ± 25.65 | 285.90 ± 37.33 | 285.53 ± 42.87 | 208.65 ± 30.24 | 349.63 ± 18.87 | 290.96 ± 40.60 | 298.38 ± 51.77 |
| ^a^Results are expressed as the mean ± SEM. | | | | | | | | | | | | |

**Table S4.** Most relevant variables detected by PLS for model 1.

| No. | Feature | VIP | Pairwise Comparison | *P*_adj_ |
| --- | --- | --- | --- | --- |
| 1 | Serum Creatinine [mg/dL] | 1.2215 | H_2_OvsPO | 8.90E-05 |
|  |  |  | H_2_OvsAPAP | 2.04E-01 |
|  |  |  | H_2_OvsCCl_4_ | 1.00E+00 |
|  |  |  | POvsAPAP | 3.74E-06 |
|  |  |  | POvsCCl_4_ | 2.45E-05 |
|  |  |  | APAPvs CCl_4_ | 1.00E+00 |
| 2 | Serum Potassium [mEq/L] | 1.3871 | H_2_OvsPO | 1.00E+00 |
|  |  |  | H_2_OvsAPAP | 4.53E-03 |
|  |  |  | H_2_OvsCCl_4_ | 8.01E-02 |
|  |  |  | POvsAPAP | 3.32E-03 |
|  |  |  | POvsCCl_4_ | 4.18E-02 |
|  |  |  | APAPvs CCl_4_ | 1.00E+00 |
| 3 | Osmolar Clearance [μL/min] | 1.0573 | H_2_OvsPO | 1.00E+00 |
|  |  |  | H_2_OvsAPAP | 8.94E-03 |
|  |  |  | H_2_OvsCCl_4_ | 1.00E+00 |
|  |  |  | POvsAPAP | 1.98E-02 |
|  |  |  | POvsCCl_4_ | 1.00E+00 |
|  |  |  | APAPvs CCl_4_ | 2.23E-01 |
| 4 | Freewater Clearance [μL/min] | 1.0663 | H_2_OvsPO | 1.00E+00 |
|  |  |  | H_2_OvsAPAP | 2.45E-03 |
|  |  |  | H_2_OvsCCl_4_ | 1.00E+00 |
|  |  |  | POvsAPAP | 6.25E-03 |
|  |  |  | POvsCCl_4_ | 1.00E+00 |
|  |  |  | APAPvs CCl_4_ | 5.03E-02 |
| 5 | PAH t/m ratio | 1.4252 | H_2_OvsPO | 2.90E-01 |
|  |  |  | H_2_OvsAPAP | 8.02E-03 |
|  |  |  | H_2_OvsCCl_4_ | 7.22E-02 |
|  |  |  | POvsAPAP | 1.00E+00 |
|  |  |  | POvsCCl_4_ | 1.00E+00 |
|  |  |  | APAPvs CCl_4_ | 1.00E+00 |
| 6 | Renal LPO [URF/mg protein] | 1.5842 | H_2_OvsPO | 1.00E+00 |
|  |  |  | H_2_OvsAPAP | 3.18E-02 |
|  |  |  | H_2_OvsCCl_4_ | 1.42E-04 |
|  |  |  | POvsAPAP | 6.18E-01 |
|  |  |  | POvsCCl_4_ | 1.02E-02 |
|  |  |  | APAPvs CCl_4_ | 4.91E-01 |
| 7 | Renal ROS [nM of DCF/mg protein/h] | 1.5304 | H_2_OvsPO | 1.00E+00 |
|  |  |  | H_2_OvsAPAP | 6.04E-04 |
|  |  |  | H_2_OvsCCl_4_ | 4.11E-02 |
|  |  |  | POvsAPAP | 6.77E-04 |
|  |  |  | POvsCCl_4_ | 2.67E-02 |
|  |  |  | APAPvs CCl_4_ | 9.93E-01 |
| 8 | Hepatic LPO [URF/mg protein] | 1.6235 | H_2_OvsPO | 9.40E-02 |
|  |  |  | H_2_OvsAPAP | 3.51E-03 |
|  |  |  | H_2_OvsCCl_4_ | 3.89E-03 |
|  |  |  | POvsAPAP | 3.87E-05 |
|  |  |  | POvsCCl_4_ | 4.23E-05 |
|  |  |  | APAPvs CCl_4_ | 1.00E+00 |
| 9 | Hepatic ROS [nM of DCF/mg protein/h] | 2.042 | H_2_OvsPO | 1.00E+00 |
|  |  |  | H_2_OvsAPAP | 3.68E-09 |
|  |  |  | H_2_OvsCCl_4_ | 3.91E-07 |
|  |  |  | POvsAPAP | 1.47E-08 |
|  |  |  | POvsCCl_4_ | 9.40E-07 |
|  |  |  | APAPvs CCl_4_ | 5.29E-01 |
| 10 | Serum ALP activity [U/L] | 1.2523 | H_2_OvsPO | 1.00E+00 |
|  |  |  | H_2_OvsAPAP | 8.40E-01 |
|  |  |  | H_2_OvsCCl_4_ | 1.87E-05 |
|  |  |  | POvsAPAP | 1.00E+00 |
|  |  |  | POvsCCl_4_ | 1.79E-04 |
|  |  |  | APAPvs CCl_4_ | 4.73E-03 |
| 11 | Serum ALT activity [U/L] | 1.2956 | H_2_OvsPO | 3.54E-01 |
|  |  |  | H_2_OvsAPAP | 5.06E-01 |
|  |  |  | H_2_OvsCCl_4_ | 1.62E-14 |
|  |  |  | POvsAPAP | 1.00E+00 |
|  |  |  | POvsCCl_4_ | 8.74E-12 |
|  |  |  | APAPvs CCl_4_ | 6.63E-12 |

**Table S5.** Most relevant variables detected by PLS for model 2.

| No. | Feature | VIP | Pairwise Comparison | *P*_adj_ |
| --- | --- | --- | --- | --- |
| 1 | GFR [mL/min] | 1.2726 | H_2_Ovs1a | 2.61E-02 |
|  |  |  | H_2_Ovs1b | 7.63E-03 |
|  |  |  | H_2_Ovs3a | 1.05E-02 |
|  |  |  | H_2_Ovs3b | 8.66E-01 |
|  |  |  | 1avs1b | 1.00E+00 |
|  |  |  | 1avs3a | 1.00E+00 |
|  |  |  | 1avs3b | 1.00E+00 |
|  |  |  | 1bvs3a | 1.00E+00 |
|  |  |  | 1bvs3b | 9.92E-01 |
|  |  |  | 3avs3b | 1.00E+00 |
| 2 | Serum Creatinine [mg/dL] | 1.2752 | H_2_Ovs1a | 1.00E+00 |
|  |  |  | H_2_Ovs1b | 8.91E-04 |
|  |  |  | H_2_Ovs3a | 3.59E-01 |
|  |  |  | H_2_Ovs3b | 1.00E+00 |
|  |  |  | 1avs1b | 1.40E-02 |
|  |  |  | 1avs3a | 1.00E+00 |
|  |  |  | 1avs3b | 1.00E+00 |
|  |  |  | 1bvs3a | 5.44E-01 |
|  |  |  | 1bvs3b | 3.18E-04 |
|  |  |  | 3avs3b | 7.44E-02 |
| 3 | Potassium Clearance [μL/min] | 1.1295 | H_2_Ovs1a | 3.46E-02 |
|  |  |  | H_2_Ovs1b | 5.83E-03 |
|  |  |  | H_2_Ovs3a | 4.38E-01 |
|  |  |  | H_2_Ovs3b | 2.37E-01 |
|  |  |  | 1avs1b | 1.00E+00 |
|  |  |  | 1avs3a | 1.00E+00 |
|  |  |  | 1avs3b | 1.00E+00 |
|  |  |  | 1bvs3a | 1.00E+00 |
|  |  |  | 1bvs3b | 1.00E+00 |
|  |  |  | 3avs3b | 1.00E+00 |
| 4 | Filtered Load of Sodium [μEq/min] | 1.2911 | H_2_Ovs1a | 1.12E-01 |
|  |  |  | H_2_Ovs1b | 1.20E-02 |
|  |  |  | H_2_Ovs3a | 1.28E-02 |
|  |  |  | H_2_Ovs3b | 8.60E-01 |
|  |  |  | 1avs1b | 1.00E+00 |
|  |  |  | 1avs3a | 1.00E+00 |
|  |  |  | 1avs3b | 1.00E+00 |
|  |  |  | 1bvs3a | 1.00E+00 |
|  |  |  | 1bvs3b | 1.00E+00 |
|  |  |  | 3avs3b | 1.00E+00 |
| 5 | Filtered Load of Glucose [mg/min] | 1.3343 | H_2_Ovs1a | 3.28E-02 |
|  |  |  | H_2_Ovs1b | 1.29E-02 |
|  |  |  | H_2_Ovs3a | 4.96E-03 |
|  |  |  | H_2_Ovs3b | 8.27E-01 |
|  |  |  | 1avs1b | 1.00E+00 |
|  |  |  | 1avs3a | 1.00E+00 |
|  |  |  | 1avs3b | 1.00E+00 |
|  |  |  | 1bvs3a | 1.00E+00 |
|  |  |  | 1bvs3b | 1.00E+00 |
|  |  |  | 3avs3b | 7.92E-01 |
| 6 | Fractional Excretion of Glucose [RAU] | 1.3343 | H_2_Ovs1a | 1.71E-01 |
|  |  |  | H_2_Ovs1b | 8.18E-03 |
|  |  |  | H_2_Ovs3a | 1.17E-02 |
|  |  |  | H_2_Ovs3b | 1.02E-01 |
|  |  |  | 1avs1b | 1.00E+00 |
|  |  |  | 1avs3a | 1.00E+00 |
|  |  |  | 1avs3b | 1.00E+00 |
|  |  |  | 1bvs3a | 1.00E+00 |
|  |  |  | 1bvs3b | 1.00E+00 |
|  |  |  | 3avs3b | 1.00E+00 |
| 7 | Freewater Clearance [μL/min] | 1.3291 | H_2_Ovs1a | 5.28E-01 |
|  |  |  | H_2_Ovs1b | 2.53E-01 |
|  |  |  | H_2_Ovs3a | 1.53E-02 |
|  |  |  | H_2_Ovs3b | 8.83E-01 |
|  |  |  | 1avs1b | 1.00E+00 |
|  |  |  | 1avs3a | 1.00E+00 |
|  |  |  | 1avs3b | 1.00E+00 |
|  |  |  | 1bvs3a | 1.00E+00 |
|  |  |  | 1bvs3b | 1.00E+00 |
|  |  |  | 3avs3b | 1.00E+00 |
| 8 | Osmolar Clearance [μL/min] | 1.2334 | H_2_Ovs1a | 1.00E+00 |
|  |  |  | H_2_Ovs1b | 1.00E+00 |
|  |  |  | H_2_Ovs3a | 3.28E-02 |
|  |  |  | H_2_Ovs3b | 1.00E+00 |
|  |  |  | 1avs1b | 1.00E+00 |
|  |  |  | 1avs3a | 1.00E+00 |
|  |  |  | 1avs3b | 1.00E+00 |
|  |  |  | 1bvs3a | 1.00E+00 |
|  |  |  | 1bvs3b | 1.00E+00 |
|  |  |  | 3avs3b | 1.00E+00 |
| 9 | PAH t/m ratio | 1.7571 | H_2_Ovs1a | 1.00E+00 |
|  |  |  | H_2_Ovs1b | 7.02E-02 |
|  |  |  | H_2_Ovs3a | 8.95E-01 |
|  |  |  | H_2_Ovs3b | 2.79E-02 |
|  |  |  | 1avs1b | 5.79E-01 |
|  |  |  | 1avs3a | 1.00E+00 |
|  |  |  | 1avs3b | 2.95E-01 |
|  |  |  | 1bvs3a | 1.00E+00 |
|  |  |  | 1bvs3b | 1.00E+00 |
|  |  |  | 3avs3b | 1.00E+00 |

**Table S6.** Most relevant variables detected by PLS for model 3.

| No. | Feature | VIP | Pairwise Comparison | *P*_adj_ |
| --- | --- | --- | --- | --- |
| 1 | Sodium Clearance [μL/min] | 1.5739 | H_2_OvsT80 | 1.00E+00 |
|  |  |  | H_2_Ovs2a | 1.00E+00 |
|  |  |  | H_2_Ovs2b | 1.87E-05 |
|  |  |  | T80vs2a | 1.00E+00 |
|  |  |  | T80vs2b | 1.15E-04 |
|  |  |  | 2avs2b | 1.89E-04 |
| 2 | Urinary Sodium Excretion [μEq/min] | 1.5606 | H_2_OvsT80 | 1.00E+00 |
|  |  |  | H_2_Ovs2a | 1.00E+00 |
|  |  |  | H_2_Ovs2b | 2.01E-05 |
|  |  |  | T80vs2a | 1.00E+00 |
|  |  |  | T80vs2b | 1.22E-04 |
|  |  |  | 2avs2b | 1.74E-04 |
| 3 | FES [RAU] | 1.1943 | H_2_OvsT80 | 1.00E+00 |
|  |  |  | H_2_Ovs2a | 1.00E+00 |
|  |  |  | H_2_Ovs2b | 1.03E-01 |
|  |  |  | T80vs2a | 5.30E-01 |
|  |  |  | T80vs2b | 1.97E-02 |
|  |  |  | 2avs2b | 9.27E-01 |
| 4 | Potassium Clearance [μL/min] | 1.1529 | H_2_OvsT80 | 1.40E-01 |
|  |  |  | H_2_Ovs2a | 1.00E+00 |
|  |  |  | H_2_Ovs2b | 6.75E-03 |
|  |  |  | T80vs2a | 1.74E-01 |
|  |  |  | T80vs2b | 1.00E+00 |
|  |  |  | 2avs2b | 1.33E-02 |
| 5 | Urinary Potassium Excretion [μEq/min] | 1.1978 | H_2_OvsT80 | 1.19E-01 |
|  |  |  | H_2_Ovs2a | 1.00E+00 |
|  |  |  | H_2_Ovs2b | 1.31E-03 |
|  |  |  | T80vs2a | 3.33E-01 |
|  |  |  | T80vs2b | 7.80E-01 |
|  |  |  | 2avs2b | 8.55E-03 |
| 6 | Filtered Load of Glucose [mg/min] | 1.0591 | H_2_OvsT80 | 6.25E-01 |
|  |  |  | H_2_Ovs2a | 1.00E+00 |
|  |  |  | H_2_Ovs2b | 6.09E-02 |
|  |  |  | T80vs2a | 2.80E-01 |
|  |  |  | T80vs2b | 1.00E+00 |
|  |  |  | 2avs2b | 3.28E-02 |
| 7 | Urinary Flow Rate [μL/min] | 1.2771 | H_2_OvsT80 | 1.00E+00 |
|  |  |  | H_2_Ovs2a | 1.00E+00 |
|  |  |  | H_2_Ovs2b | 3.08E-02 |
|  |  |  | T80vs2a | 1.00E+00 |
|  |  |  | T80vs2b | 6.15E-01 |
|  |  |  | 2avs2b | 7.05E-01 |
| 8 | Freewater Clearance [μL/min] | 1.2792 | H_2_OvsT80 | 1.00E+00 |
|  |  |  | H_2_Ovs2a | 1.00E+00 |
|  |  |  | H_2_Ovs2b | 1.20E-02 |
|  |  |  | T80vs2a | 1.00E+00 |
|  |  |  | T80vs2b | 3.20E-01 |
|  |  |  | 2avs2b | 1.88E-01 |
| 9 | Osmolar Clearance [μL/min] | 1.3139 | H_2_OvsT80 | 1.00E+00 |
|  |  |  | H_2_Ovs2a | 1.00E+00 |
|  |  |  | H_2_Ovs2b | 1.20E-02 |
|  |  |  | T80vs2a | 1.00E+00 |
|  |  |  | T80vs2b | 3.46E-01 |
|  |  |  | 2avs2b | 2.55E-01 |
| 10 | PAH t/m ratio | 1.7911 | H_2_OvsT80 | 3.00E-01 |
|  |  |  | H_2_Ovs2a | 5.29E-05 |
|  |  |  | H_2_Ovs2b | 2.53E-01 |
|  |  |  | T80vs2a | 3.61E-02 |
|  |  |  | T80vs2b | 1.00E+00 |
|  |  |  | 2avs2b | 4.28E-02 |
| 11 | Renal LPO [URF/mg protein] | 1.3374 | H_2_OvsT80 | 8.78E-01 |
|  |  |  | H_2_Ovs2a | 1.00E+00 |
|  |  |  | H_2_Ovs2b | 4.85E-03 |
|  |  |  | T80vs2a | 1.00E+00 |
|  |  |  | T80vs2b | 3.47E-01 |
|  |  |  | 2avs2b | 1.26E-01 |
| 12 | Serum ALP activity [U/L] | 1.1342 | H_2_OvsT80 | 1.00E+00 |
|  |  |  | H_2_Ovs2a | 1.00E+00 |
|  |  |  | H_2_Ovs2b | 1.57E-02 |
|  |  |  | T80vs2a | 1.00E+00 |
|  |  |  | T80vs2b | 4.81E-01 |
|  |  |  | 2avs2b | 1.17E-01 |
| 13 | Serum ALT activity [U/L] | 1.001 | H_2_OvsT80 | 2.33E-02 |
|  |  |  | H_2_Ovs2a | 3.47E-01 |
|  |  |  | H_2_Ovs2b | 1.39E-01 |
|  |  |  | T80vs2a | 1.00E+00 |
|  |  |  | T80vs2b | 1.00E+00 |
|  |  |  | 2avs2b | 1.00E+00 |

**Table S7.** Most relevant variables detected by PLS for model 4.

| No. | Feature | VIP | Pairwise Comparison | *P*_adj_ |
| --- | --- | --- | --- | --- |
| 1 | Serum Glucose [mg/dL] | 1.1005 | H_2_OvsPO | 2.46E-01 |
|  |  |  | H_2_Ovs4 | 3.31E-01 |
|  |  |  | POvs4 | 1.93E-02 |
| 2 | Fractional Excretion of Glucose [RAU] | 1.0344 | H_2_OvsPO | 1.87E-01 |
|  |  |  | H_2_Ovs4 | 2.51E-01 |
|  |  |  | POvs4 | 1.08E-02 |
| 3 | Urinary Sodium Excretion [μEq/min] | 1.4603 | H_2_OvsPO | 3.21E-01 |
|  |  |  | H_2_Ovs4 | 4.96E-02 |
|  |  |  | POvs4 | 1.00E+00 |
| 4 | PAH t/m ratio | 1.88 | H_2_OvsPO | 1.97E-01 |
|  |  |  | H_2_Ovs4 | 1.16E-02 |
|  |  |  | POvs4 | 8.17E-01 |
| 5 | Renal LPO [URF/mg protein] | 2.0758 | H_2_OvsPO | 1.00E+00 |
|  |  |  | H_2_Ovs4 | 2.17E-03 |
|  |  |  | POvs4 | 5.00E-02 |
| 6 | Serum ALT activity [U/L] | 1.6581 | H_2_OvsPO | 1.59E-01 |
|  |  |  | H_2_Ovs4 | 2.35E-02 |
|  |  |  | POvs4 | 1.00E+00 |

**Table S8.** Most relevant variables detected by PLS for model 5.

| No. | Feature | VIP | Pairwise Comparison | *P*_adj_ |
| --- | --- | --- | --- | --- |
| 1 | GFR [mL/min] | 1.1638 | H_2_OvsPO | 1.37E-01 |
|  |  |  | H_2_OvsT80 | 1.11E-01 |
|  |  |  | POvsT80 | 3.39E-03 |
| 2 | Serum Creatinine [mg/dL] | 2.0256 | H_2_OvsPO | 1.55E-04 |
|  |  |  | H_2_OvsT80 | 1.00E+00 |
|  |  |  | POvsT80 | 1.15E-04 |
| 3 | Filtered Load of Sodium [μEq/min] | 1.1465 | H_2_OvsPO | 1.36E-01 |
|  |  |  | H_2_OvsT80 | 1.36E-01 |
|  |  |  | POvsT80 | 4.13E-03 |
| 4 | Urinary Potassium Excretion [μEq/min] | 1.0512 | H_2_OvsPO | 1.00E+00 |
|  |  |  | H_2_OvsT80 | 4.10E-02 |
|  |  |  | POvsT80 | 1.79E-01 |
| 5 | Fractional Excretion of Potassium [RAU] | 2.0279 | H_2_OvsPO | 1.05E-03 |
|  |  |  | H_2_OvsT80 | 1.00E+00 |
|  |  |  | POvsT80 | 3.79E-03 |
| 6 | Filtered Load of Potassium [μEq/min] | 1.0932 | H_2_OvsPO | 1.98E-01 |
|  |  |  | H_2_OvsT80 | 1.38E-01 |
|  |  |  | POvsT80 | 6.17E-03 |
| 7 | Filtered Load of Glucose [mg/min] | 1.1591 | H_2_OvsPO | 9.17E-02 |
|  |  |  | H_2_OvsT80 | 2.19E-01 |
|  |  |  | POvsT80 | 4.57E-03 |
| 8 | Hepatic LPO [URF/mg protein] | 1.5596 | H_2_OvsPO | 1.53E-02 |
|  |  |  | H_2_OvsT80 | 1.00E+00 |
|  |  |  | POvsT80 | 9.01E-03 |
| 9 | Serum ALT activity [U/L] | 1.3891 | H_2_OvsPO | 2.78E-01 |
|  |  |  | H_2_OvsT80 | 2.86E-02 |
|  |  |  | POvsT80 | 1.00E+00 |
